# HIV knowledge and sexual risk behavior among street adolescents in rehabilitation centres in Kinshasa; DRC: gender differences

**DOI:** 10.4314/pamj.v10i0.72233

**Published:** 2011-10-17

**Authors:** Albert Mudingayi, Prosper Lutala, Bavon Mupenda

**Affiliations:** 1 Reproductive Health Centre for Youths and Teenagers Bomoto. Christian Church in Congo, Kinshasa, DRC; 2 Département de Médecine de Famille, Faculté de Médecine, Université de Goma, République Démocratique du Congo; 3 Centre Interdisciplinaire de Bioéthique pour l'Afrique Francophone, Ecole de santé publique, Faculté de Médecine, Université de Kinshasa, République Démocratique du Congo

**Keywords:** Condoms, Knowledge, Sexual risk behavior, street children, Kinshasa, Congo

## Abstract

**Background:**

Street children, common in Africa, are increasingly vulnerable to alcohol and drugs of abuse and lack access to both healthcare and knowledge about HIV and AIDS. Hence, this study assessed the level of knowledge about sexually transmitted infections (STIs), including HIV, among street adolescents in the Democratic Republic of the Congo (DRC).

**Methods:**

A random sampling of 200 street children (10-25 years of age) were selected from 17 rehabilitation centres in Kinshasa, and a structured questionnaire was administered to all participants in their respective centres. High knowledge, knowledge or awareness of condom was defined when a participant gave more than 67% of correct responses. Chi square analysis was used to test differences between sexes.

**Results:**

The knowledge level of respondents was high. 54.3% of males and 45.7% of girls have heard about HIV), and few participants cited unprotected sex as mode of transmission (42.9% for males and 57.1% for females). A high number of children reported a previous sexual experience. Satisfying a natural bodily need was the main reason for having sex. However, the use of condoms is still low in both genders (26.2 versus 59.3%, p<0.01). Neither gender reported a reason why they are not using a condom.

**Conclusion:**

This study highlights the high knowledge about HIV, which contrasts with low condom use and high past sexual experiences with the high number of sexual partners and sexual contacts. Policies targeting these findings are warranted to reverse such trends.

## Background

Sub-Saharan Africa, including the Democratic Republic of the Congo (DRC), has been hit hard by HIV and currently accounts for more than two-thirds of recent HIV infections worldwide [1]. DRC has approximately 1,034,086 infected people (>15 years old) with 241,452 of those in need of antiretroviral therapy (ART) [2]. This high rate of HIV infection is fueled by a complex emergency situation [3] embattling the country, which is comprised of internal conflict with large-scale displacements of people, food shortages, and fragile economic, political, and social institutions. This crisis led to an increased number of individuals infected with HIV, including children. Furthermore, the toll number of deaths from some preventable diseases is increasing leading to high number of orphans. High vulnerability of those orphans and other children due to this complex emergency state turns some of them into streets, whose numbers are increasing in DRC with 2008 estimates reaching 20,000 in Kinshasa [4].

Along with some structural factors like poverty, psychosocial correlates (including norms, beliefs and attitudes); knowledge plays a role as a predictor of HIV risk behaviour [5]. Although several factors play a role on HIV risk behavior, HIV knowledge remains the key modifiable factors [5 , 6].

Knowledge about HIV-related issues has been shown to be associated with delayed onset of sexual relations [7], consistent use of condoms, reduction in the number of sexual partners [8], and increased tolerance for people with HIV/AIDS [9]. On the other side, lack of knowledge can lead to several problems. It was found for example that lack of correct information about pathways of HIV transmission may contribute to fewer people being tested, misperceptions about one′s level of risk, and increased likelihood of AIDS optimism, denial, and stigmatization, among others [10]. Imparting knowledge to people is the first step in developing a successful fight against any condition with a behavior-change component, like HIV/AIDS. Nonetheless, accurate knowledge on HIV/AIDS is necessary; but, by no means a sufficient condition for the consistent adoption of protective behaviors.

Several studies have shown, in developed countries a relationship between educational attainment and HIV/AIDS knowledge [11]. Students in lower status education types (e.g., vocational education) have poorer HIV/AIDS knowledge as they were pupils in lower classes; and both display high risk behavior [12 , 13]. Furthermore in Malaysia, it was found that levels of knowledge were higher (more than 90%) among adolescents enrolled in school compared to adolescents who had left school [14].

In spite of satisfactory level of HIV knowledge in youth found in several parts of the world, studies have also found that youths significantly and concurrently report accurate and inaccurate information regarding HIV; as attested by youths in Cameroon like other settings in sub-Saharan Africa (SSA) who still have a mixture of beliefs and misconceptions about HIV transmission and prevention [15 , 16].

Street children are exposed to other threats, including sexually transmitted infections (STIs), HIV [17 , 18], alcohol and drug abuse [19 , 20], a lack of knowledge of STI prevention, and little access to healthcare [21 , 22]. Some are also exposed to risky practices, such as having multiple sexual partners, engaging in sex while under the influence of alcohol and other drugs, and exchanging sex for money [23].

Several studies have been carried out in Africa regarding knowledge about and attitudes toward HIV and STIs in youth and street children, but the common point of those studies is the lack of consistent findings across adolescents in Sub-Saharan Africa studies.

Thabo et al. [24] found that most respondents have good awareness about HIV/AIDS in Botswana. In Kinshasa, Kayembe et al. [4] found that most participants were sexually experienced and had first sexual intercourse when living on the streets. This study also demonstrated that their knowledge of the ABC prevention approach means was moderate. Condom use was the most common means of prevention. Misconceptions about HIV transmission were common, third of the participants reported that HIV could be transmitted through a mosquito bite or through witchcraft.

Lema and al. [25] in Kibaha district (Tanzania) found among young that the majority (98.4%) of the respondents have heard of HIV/AIDS. About three quarters (74.8%) of the respondents knew where to get HIV testing services but only a small proportion (28.9%) have been tested for HIV infection. Of those not yet tested, 38.2% admitted that they were ready to do so.

Secondary students from Nigeria [26] were aware that HIV could be transmitted via unprotected sex; contrary to young migrants in Nigeria [27], secondary students in Benin City, Nigeria [28], and female children in Accra, Ghana [29].

Several determinants of HIV knowledge were identified from several studies. Studies have demonstrated that AIDS knowledge is associated with condom use. Low level of knowledge about the transmission and prevention of AIDS among adolescents was a predictor of non-use of Condoms [30]. The HIV knowledge was also, in a logistic regression model correlate to age, education level, socio-economical level/profession of the mother, sexual practices among others [4,31]

Shelter children in Kinshasa are selected from street following some set criteria, specific to each center. After admission, depending on age and ability some are sent to formal school to continue their education while a second is recruited for vocational training in different fields to acquire some skills. However, both groups are theoretically trained, during free times and holidays on sexual education to prevent STIs including HIV-AIDS and unplanned pregnancies [4]. While people are used to the concept of street children in Congo, there is a new approach of their management called sheltered children which is yet to be documented. Therefore, we determined the STI-HIV knowledge of street children in a rehabilitation centre, their sexual behaviors, and the determinants of their HIV-STI knowledge.

## Methods

The present study was descriptive and cross-sectional, and used a structured questionnaire. The present study was conducted in March 2005 in Kinshasa, DRC.

The study population was composed of youth living in 50 rehabilitation centres, collectively called “Réseau des Éducateurs des Jeunes et Enfants de la Rue.” Stratified random sampling of 17 centres was conducted from an alphabetical list of all 50 sites, from which respondents were drawn. From the list, all who consented to participate and those without psychological deficits were maintained. Due to financial and time constraints, a convenience sample of 200 participants were selected by considering sex as our main criteria (108 males and 96 females, representing 54% males and 46% females in the population). We excluded from the study those participants younger than 10 years of age.

A team of four trained field workers was selected and distributed the questionnaires after explaining the content to the participants. The final version of the questionnaire, translated into Lingala, the local language, was distributed to all selected participants. Illiterate participants or those unable to write easily were assisted in completing the forms by literate peers selected among those who were excluded from the study. Street children are becoming a veritable threat for Kinshasa population and are involved in stealing, gangs activities. This has undermined their relations with the general public and in case of problem in public places, they are the first suspected and arrested. On themselves they have reacted by a retraction in most of events going on in the city. For the sake of empowering them, increase the trust in us and avoid them feeling like responding to police questionnaire or to “lawyer's questions” during the interview/explanation, we opted to this self-administration as suggested by another study [32].

The questionnaire, adapted from previous studies [31,33,34] collected data on socio-demographic data (age, sex, formal education, current activities or occupation including schooling); sexual practices; knowledge of HIV- AIDS; knowledge and use of condoms and self-perceived risks of STIs and AIDS. The Items used for HIV Knowledge, condom use variables can be found in Box 1 (additional material). Cronbach's alpha for the AIDS knowledge scale was 0.68. HIV knowledge and use of condom scores were computed from the correct responses to the current interview. Condom use was adapted from the WHO AIDS KAP conducted in Ghana and the study by Diclemente [31,34]. The first have a reliability Cronbach's alpha of 0.74. Original Knowledge questionnaire from above study [34] had a Cronbach's alpha of .68.

Knowledge/awareness of HIV/STI was defined as the number of correct answers to 75% of questions in each subgroup of questions (i.e., general information on HIV, modes of transmission, and symptoms of STI/HIV). Response options were closed: either yes, no, or “I don't know” and were scored for the number of correct responses, with “I don′t know” scored as incorrect. Scores were coded into three levels: no or low knowledge=0 to 33% correct responses; medium knowledge=correct answers between 33-67% correct responses; and high knowledge, knowledge or awareness of condom=more than 67% correct responses. Thereafter, the scores were collapsed into two categories. Those with low and medium scores were categorized as absence of knowledge and the remainders were considered knowledge or awareness of condom. The validity of the questionnaire showed Cronbach's alpha of 0.9246 and 0.8287 (data not shown) for condom use and HIV/AIDS subscales respectively.

To assess HIV risk behavior, participants indicated any previous sexual experience, one or more reason(s) for having sex, history of sexual assault, number of sexual partners, and frequency of sex during the last 30 days. All responses indicated the occurrence or non-occurrence of each risk factor for closed questions. The remaining questions were open-ended, and allowed participants to answer as desired without limit and us to deep our understanding.

The last group of questions related to condom use as described in the questionnaire. The scoring was similar to that described for knowledge of STIs/HIV.

Data analysis was performed using Statistical Package for Social Sciences (SPSS 16.0 for Windows, SPSS Inc., Chicago, IL) software. We obtained estimates of knowledge of HIV, sexual risk behavior, and condom use. Bivariate Chi square analysis was used to compare different characteristics between females and males. We also conducted logistic regression analysis to estimate the association between relevant predictor variables and knowledge of HIV. The predictor variables (condom use, frequency of sexual intercourse, hear about condom, profession/occupation, education attainment, age) were identified from the literature as possible factors that may be associated with knowledge of HIV [4,34–36].

We report adjusted odds ratios for selected predictor variables while considering knowledge of HIV status separately for males and females as dependent variables. The level of significance was set at a probability less than or equal to 0.05. The odds ratio and two-sided 95% confidence intervals (CIs) are reported. If the confidence interval does not overlap zero, the effect is said to be statistically significant.

Scales were created for the AIDS knowledge, psychosocial, and risk-taking variables by summing up all items to derive a scale score. Cronbach's alpha, ranges of scores and means, variances and correlations were computed for each of the 2 scales (data not shown).

## Results

### Sample characteristics

Of 108 (54%) males and 92 (46%) females, the median age was 14 years (IQR: 10-16) for males and 17 (IQR: 14-25) for females. 57 (52.7%) males completed primary school, while 74 (80.4%) females attended secondary school (Table 1). Females are significantly older and more highly educated.

**Table 1:** Socio-demographic baseline data of street kids involved in the study

Variables	Males(108) N (%)	Girls(92) N (%)	χ2	p
**Age ranges ( years)**				
15-19	42 (38.8)	18(19.5)	46.6	0.000
20-25	59 (54.6)	29(31.5)		
20-25	7 (6.4)	45(48.9)		
**Education level**				
Never attended school	3 (2.7)	1 (1)	27.1	0.000
Primary	57 (52.7)	17(18.)		
Secondary	48 (44.4)	74(60.4)		
**Occupation**				
Schooling	91 (84.2)	67(72.)	16.1	0.003
Formal work	0	1 (1)		
Small businesses	3 (2.7)	3 (3.2)		
Changing/instable short jobs	10 (9.2)	3 (3.2)		
Others ****	4 (3.7)	18(19.5)		

****: One or combined activities: Stealing, sweeping the market and surroundings, begging and car's cleaning, digging tombs

### Knowledge of HIV-AIDS

Knowledge of HIV is high with no differences with respect to gender (Table 2; χ2=0.05, df=1, p=0.83). The most common mode of transmission in males is by contacts with urine/stool (30.5%) versus unprotected sex (36.7%) in females. Unprotected sex was given as a means of transmission in 22.2% of males and 34.7% of females, but there were no differences between modes of transmission among these groups (χ2=10.3, df=5, p= 0.068). Ulcers and swelling on genitalia were the most common symptoms of STIs in males and females, respectively, but these differences were not statistically significant (χ2=8.13, df=6, p=0.229).

**Table 2:** HIV-STI Knowledge by sex and school level among street adolescents in rehabilitation centres in Kinshasa, DRC

Sex of participants			Boys			Girls			x2	p
**Education level**	NA	P	S	Total	NA	P	S*	Total		
**Hear of HIV**										
Yes	0	47	47	94(54.3)	1	7	71	79(45.7)	0.05	0.832
No	3	10	1	14(12.9)	0	10	3	13(14.1)		
										
**Transmission modes**										
Unprotected sex	3	12	9	24(42.9)	1	4	27	32(57.1)	10.3	0.068
Sharp objects	0	5	17	22(20.3)	0	0	6	6(6.5)		
Witchcraft	0	7	7	14(12.9)	0	2	4	6(6.5)		
Cont. Object	0	2	3	5(4.6)	0	0	9	9(9.7)		
Ur.stool contact	0	21	7	28(25.9)	0	8	14	22(23.9)		
Others **	0	10	5	15(13.8)	0	3	14	17(18.4)		
										
**Symptoms**										
Low abd. Pain	0	7	5	12(11.1)	0	1	6	7(7.6)	8.13	0.229
Painful urines	1	8	3	12(11.1)	0	1	21	22(23.9)		
White discharge	0	1	3	4(24)	0	1	7	8(8.6)		
G.Ulcers	1	5	20	26(24)	1	3	20	24(26)		
Headache	0	13	2	15(13.80	0	6	3	9(9.7)		
Swelling genitalia	0	13	13	26(24)	1	6	12	19(20.6)		
Others***	1	10	2	13(12)	0	1	10	11(11.9)		

NA: not attended school, P: primary school level; S: secondary school level. S*: some secondary school participants gave several answers; Ur. Stools contact: contact with urines and/or stools; low abd. Pain: low abdominal pains, G. Ulcer: ulcers on genitalia

**: Hands washing using the same water, by coughing, no way, by eye's contacts and skin contacts during sweating

***: Fouls-smelling from mouth, yellowish eyes, giving birth to twins more than once and frequent hiccups.

### General sexual risk for respondents

As shown in Table 3 among those who reported a history of sex, 36.1% are males and 66.3% are females and within groups comparison shows that 36.1% and 66.3% of all males and females respectively have reported a history of sex. Among those with sexual experiences, the reasons for sex varied by gender: enjoyment was the most common cause for males (74.3%) and peer pressure for females (56.4%). Sexual assault is reported in both groups: 6.8% in males and 15.2% in females. Males and females reported having 1-5 sexual partners in 69.2% and 53.8% of cases, respectively. Having one sexual experience in the last 30 days was reported in 15.3% of males and 20.5% of females. However, 74.3% of males and 61.5% of females did not disclose a number of sexual experiences in the past 30 days. These findings were not statistically significant (x2=6.63, df=4, p=0.25).

**Table 3:** Risk sexual behavior of street adolescents in rehabilitation centres in Kinshasa, DRC

Variables	Boys N (%)	Girls N (%)	χ2	p-values
**History of sexual intercourse**				
Yes	39 (36.1)	61 (66.3)	18.1	0.000
No	69 (63.8)	31 (33.6)		
				
**Reasons of sexual intercourse** (n:39)				
Respond to a need of the body	29 (74.3)	17 (43.5)	26.5	0.000
Need of money	0	7 (17.9)		
For conception	0	1 (2.5)		
Conformity	0	4 (10.2)		
Peer pressure	8 (20.5)	22 (56.4)		
Imitate pornography	81(2.5)	0		
No position	1 (2.5)	10 (25.6)		
				
**History of sexual assault**	(n: 108)	(n: 92)		
Yes	10 (6.8)	14 (15.2)	6.05	0.08
No	49 (46.5)	37 (40.2)		
No position	49 (46.5)	41 (44.5)		
				
**Number of sexual partners** (n:39)				
1 -5	27 (69.2)	21 (53.8)	6.63	0.25
6-10	4 (10.2)	4 (10.2)		
11-15	0	2 (5.1)		
16-20	0	1 (2.1)		
21-25	2 (5.1)	7 (17.9)		
Don't know	5 (12.8)	4 (10.2)		
				
**Number of sex the last 30 days**				
3-5 times	1 (2.5)	4 (10.2)	2.56	0.46
1-2 times	3 (7.6)	3 (7.6)		
Once	6 (15.3)	8 (20.5)		
Don't know	29 (74.3)	24 (61.5)		

### Sexual risk related to condom use

As shown in Table 4 91.6% of males and 85.1% of females have heard about condoms previously. However, in ‘other reasons’ for using condom (Table 5) there exist some ‘misconceptions’ in condom use (21.7% of males and 15.9% of females). Among participants, 26.2% of males and 59.3% of females have used a condom, with a significant difference (x2=20.7, df=1, p=0). Condom use is not understood by 56.5% of males and 25.5% of females. Reasons for not using a condom significantly differed between males and females (x2=20.9, p=0.004). Reasons for using a condom are nearly the same in males and females, and not statistically significant (x2=2.83, p=0.243).

**Table 4:** Use of condom among street adolescents in rehabilitation centres in Kinshasa, DRC

Variables	Boys N (%)	Girls N (%)	Chi-square	p-values
**Hear about condoms**				
Yes	99 (91.6)	86 (85.1)	0.235	0.628
No	9 (8.3)	6 (6.9)		
				
**Have you used a condom before?**				
Yes	26 (26.3)	51 (59.4)	20.7	0.000***
No	73 (73.7)	35 (40.6)		
				
**Barriers in condom's use**				
Sin	3 (3)	7 (8.1)	20.9	0.004**
Unavailability of condoms	13 (13.1)	13 (15.1)		
Expensive	3 (3)	6 (6.9)		
Decrease pleasure during sex	3 (3)	6 (6.9)		
Partner's opposition	3 (3)	3 (3.4)		
don't like condom	15 (15.1)	21 (24.4)		
Not conversant with its use	56 (56.5)	22 (25.5)		
Having one partner for so long	3 (3)	8 (9.3)		
				
**Reasons for using condom**				
Avoid STI's and HIV	49 (49.4)	37 (42)	2.83	0.243
Avoid pregnancy	30 (30.3)	37 (42)		
Others reasons ^1^	20 (21.7)	14 (15.9)		

^1^Prevent cancer, impotence, excess of pleasure, avoid dry girl, strong attachment each other

**: p< 0.01

***: p< 0.0001

**Table 5:** Determinants of STI/HIV knowledge among street adolescents in rehabilitation centres in Kinshasa, DRC

		Males				Females			
	
Variables				95% CI*, limits				95% CI, limits	
	
		n	AOR^c^	Lower	Upper	n	AOR	Lower	Upper
**Age ranges**									
	10 to 14	42	1			18	1		
	15 to 15	59	0.996	0.8769	0.9869	29	0.745	1.867	0.954
	20 to 25	7	1.765	1.6543	3.5436	45	1.32	1.005	2.328
**Education**									
	Illiterate	3	1			1	1		
	Primary	57	3,666	1.654	8.125	17	1.801	0.213	4.213
	Secondary	58	1,767	0.796	3.924	74	5.086	1.768	7.432
**Occupation**									
	Schooling	91	3,871	0.906	16.538	67	1.593	0.474	5.353
	Others	0	1			1	1		
**HAH****^b^**									
	Yes	94	1,623	0.494	5.312	79	0.715	0.144	0.682
	No	14	1			13	1		
**NSP****^a^**									
	1 to 5	39	0,322	0.1403	0.7399	21	2.001	0.459	8.698
	>5	69	1			18	1		
**Frequency of sex**									
	≤5	27	1.275	0.235	6.899	21	2.553	0.7003	9.3113
	≥6	12	1			18	1		
**Hear about condoms**									
	Yes	99	4.827	1.1302	20.6207	86	5.468	1.318	22.683
	No	9	1			6	1		
**Use condoms**									
	Yes	26	4.791	0.9277	24.7507	51	1.077	0.383	3.028
	No	13	1			35	1		

^b^: Hear about HIV

^a^: Number of sexual partners

^c^: adjusted odds-ratio

*: 95% confidence intervals.

The results of the logistic regression analysis are displayed in Table 5. In males, HIV knowledge correlates with an older age range, having 1 to 5 sexual partners, and hearing about condoms. Children between 19 and 25 years of age are almost twice as knowledgeable as teenagers (OR= 1.765, 95% CI 1.554-3.543). Teenagers between 15-19 years with 0-5 sexual partners have less HIV knowledge (OR= 0.996; 95% CI 0.876- 0.986) than their counterparts with more than five sexual partners (OR= 0.322; 95% CI 0140-0.739). Those hearing about condoms are four times more likely to have HIV knowledge than all others (OR: 4.827; 95% CI 1.130-20.620). In females, HIV knowledge correlates with an older age range, attaining primary or secondary education, hearing about HIV, and hearing about condoms.

In females, older participants are more likely than others to have good HIV knowledge (OR: 1.32; 95% CI 1.005-2.328) while those with a secondary education level are five times more likely to demonstrate good HIV knowledge than those who are illiterate (OR= 5.086, 95% CI 1.768-7.432) or those who have heard about condoms (OR= 5.468, CI 1.318- 22.683).

## Discussion

### Strengths and weaknesses of the study

The present study extends prior evidence by examining the STI-HIV knowledge level and risky behaviors among street children living in rehabilitation centres in Kinshasa, where sexual behaviors are expected to be different than those of “pure street children”. The fact that these centres were selected in different areas of the city increase the likelihood of diverse views as far as sexual issues are concerned.

Gender was recognized in previous sexual studies as a main confounder, and results stratified accordingly. However, those results must be interpreted in light of several limitations:

The cross-sectional design of the current study prohibits inference of causality in any way. A child might report using a condom in a past sexual experience to receive praise or self-satisfaction. Social desirability compels participants to over-report good sexual behaviors and higher condom use. Also, sexual issues are very sensitive and could limit free expression in some matters.

The self-administered questionnaire lacks power to detect all misunderstandings despite the presence of a researcher/interviewer in the field. Self-reported assessments of sexual behaviour are prone to a number of biases that could affect the reliability and validity of a measure such as participant's literacy level and comprehension of behavioural terminology, recall biases and self-presentation or confidentiality concerns resulting from stigmatization of the behaviour in question [37].

To minimise the effect of this we ensured full confidentiality of participants, a research assistant was present in the room to respond to possible questions raised by participants during the data collection, and also we reduce our questionnaire to be as simple as possible. The questions were asked directly without any hesitations and starting by the less embarrassing, validation of the instrument was done from our sample, and during the whole process we were sensible to contextual issues of the city (Kinshasa), the street children and of each shelter. Finally, we established a working trust with questionnaire respondents by including among us some of their supervisors, known by them but working in sisters centres [37].

Inadequate sampling and the small sample size restrain the generalization of the findings to the whole population of street children living in rehabilitation centres. However, the proportions stratified by gender and education attainment have minimized this worry. Validation of the tool used in the adapted questionnaires were conducted prior to data collection and could, to some extent, lead to misconceptions from respondents. However, one of the instruments was adapted from an instrument used in Ghana.

Current HIV knowledge questionnaires are expert-based, omitting beliefs underlying superficially correct knowledge. Therefore, these results do not reflect what young people need to know to address their risk behaviors [38] based on possible misconceptions which could explain the discrepancy in the reports of high HIV knowledge and high-risk behaviours among participants [39].

### HIV knowledge and high-risk behaviors among participants

Despite these limitations, this paper provides current evidence of street children living in rehabilitation centres in Kinshasa and confirms previous studies. We found low knowledge of HIV-STI in street children, high education levels of females compared to males, a high condom use rate, high numbers of sexual partners, and common misconceptions.

### Strengths and weaknesses relative to other studies

The fact that females are older is difficult to explain. Street females in another study [40] left their families due to intra-family conflicts and abuse, delaying family reintegration.

That some modes of transmission of HIV were either given by few participants (unprotected sex) or none at all (mother to child transmission) needs further reflection. This appears contrary to HIV knowledge, which is defined as having the ability to recall facts concerning causes, transmission, and prevention [24]. Low knowledge about the correct modes of transmission undermines the use of condoms as elsewhere [28,41].

Despite awareness of condom and reasons for using condoms, problems still exist regarding their use. Higher female condom use agrees with previous research [19], but should be interpreted with caution, as use seems context-dependent [22].

Misconceptions in the mode of transmission (Table 2) are of great concern. For example, hand washing, physical contact, and witchcraft were mistakenly listed by participants as modes of HIV transmission. From an educational perspective, these misconceptions can easily lead to stigmatization of infected people. The belief in witchcraft as a mode of HIV transmission confirms previous study [4].

Our study agrees with a study conducted in Uganda that showed the main reasons for reckless sexual behaviors was seek of sexual pleasure for males, and seek for money in females [23]. The high number of sexual partners and high frequency of sex in females also corroborates with previous evidences [4,42].

Regarding condom use, the greater age of the females, the increased numbers of sexual experiences, the fact that condoms are used for contraception and STI prevention, and their perceived vulnerability to HIV play a role in their high rates of condom use. This is a good sign, considering that knowledge of condom use by adolescent females has been associated with a decreased number of sexual partners [9].

The current study showed that despite comprehensive HIV education given to children in rehabilitation centres, a high level of knowledge does not translate into lower rates of risky sexual behaviors and question the efficacy of some HIV health interventions in Sub-Saharan countries [42–46]; and, only community-wide risk reduction programs [23] appear to be effective. Further studies with more elaborate designs, such as blind, randomized, controlled trials or biomedical end-point studies [41] that assess objective outcomes could provide more validity on reported sexual behaviors. Research questions about prevalence/incidence of HIV and other STIs, or pregnancies (biomedical end-points studies) could shed more light on condom use compared to the self-reported use of condoms.

In line with past evidences [4]; HIV/STI knowledge, increased age, enrollment in secondary school, and hearing about condoms correlate with high HIV knowledge. As stated in the literature, knowledge is associated with a greater use of condoms. Also, an association with education supports high condom use. Some explanations given above are still supported by these associations.

### Meaning of the study

This article illustrates that HIV-STI preventive measures can aid street children irrespective of their location. This observation is supported by similarities in knowledge level, sexual risk behavior, and condom use.

### Unanswered questions and future research

Roles of some others variables (sexual history, past family history, characteristics of rehabilitations centres as others HIV-knowledge determinants) still unexplored in the rehabilitation centres in Kinshasa.

## Conclusion

Built on previous reports regarding condom use and sexual practices among street children, this study examined children living in rehabilitation centres in Kinshasa, DRC, highlighting deficiencies with regard to HIV transmission routes, knowledge of symptoms, number of sexual partners, frequency of sexual intercourse, use of condoms, and past experience of sexual assault. Correlates of HIV knowledge in the shelters were identified.

### Implications of the study

Education and the integration of street children must include these above-mentioned deficiencies in the content of their school curriculum to increase their knowledge and reduce their vulnerability for HIV/STIs for their full participation in the development of their country.

Horizontal research coupled to the sensitization of street children in rehabilitation settings and the determination of other sexual parameters must be undertaken and cross-checked using analytical approaches to guide actions based on results. Studies on biomedical end-points and knowledge, behaviors, and practices should be done to bridge the gap between good knowledge of HIV and risk behaviors found in children.

## Additional material

Multi-item statements to measure HIV-STI Knowledge of participants and condom use (PDF 56 Ko)
